# Supplementary material for: Depth and temperature preferences of meagre, Argyrosomus regius, as revealed by satellite telemetry
Source: PLoS One. 2023 Nov 21;18(11):e0288706. doi: 10.1371/journal.pone.0288706 (PMC10662727; doi:10.1371/journal.pone.0288706)
Supplement: S1 Fig — Dashed lines indicate crepuscular hours around the study site. (PDF) [file pone.0288706.s001.pdf]

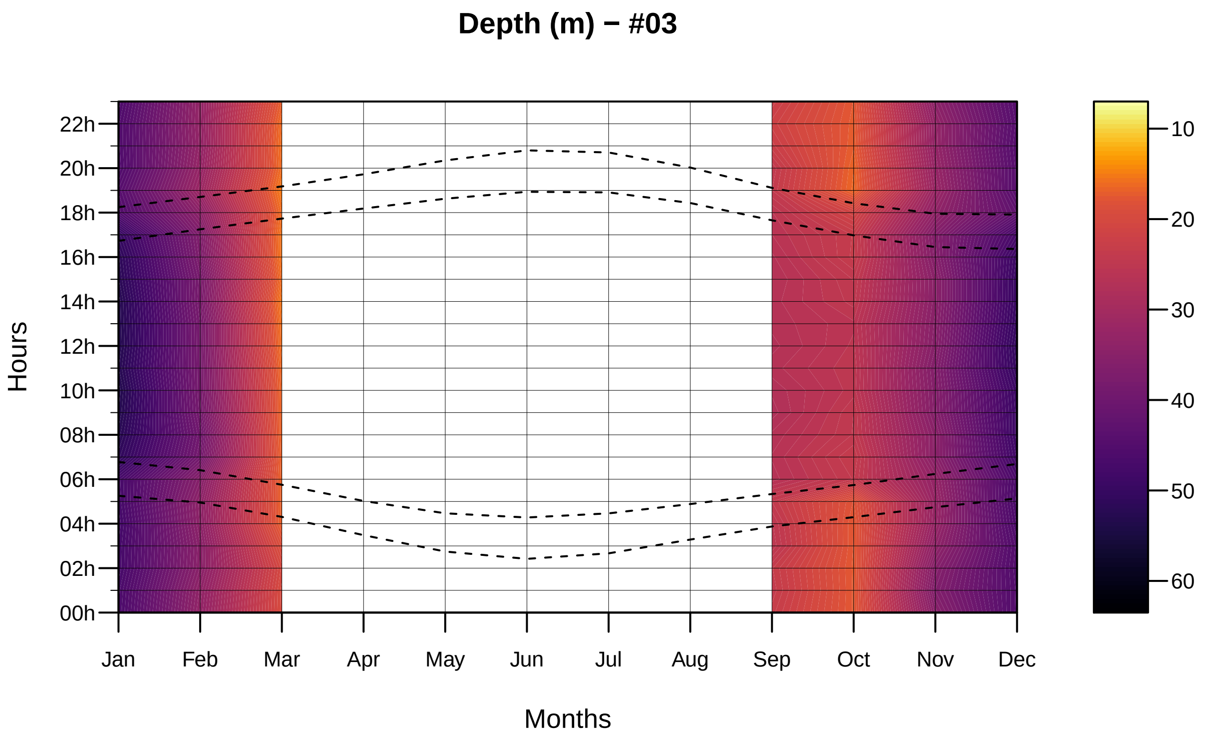

Depth (m) − #03
22h
20h
18h
16h
14h
12h
10h
08h
06h
04h
02h
00h
Hours
Jan
Feb
Mar
Apr
May
Jun
Jul
Aug
Sep
Oct
Nov
Dec
Months
10
20
30
40
50
60

Depth (m) − #04

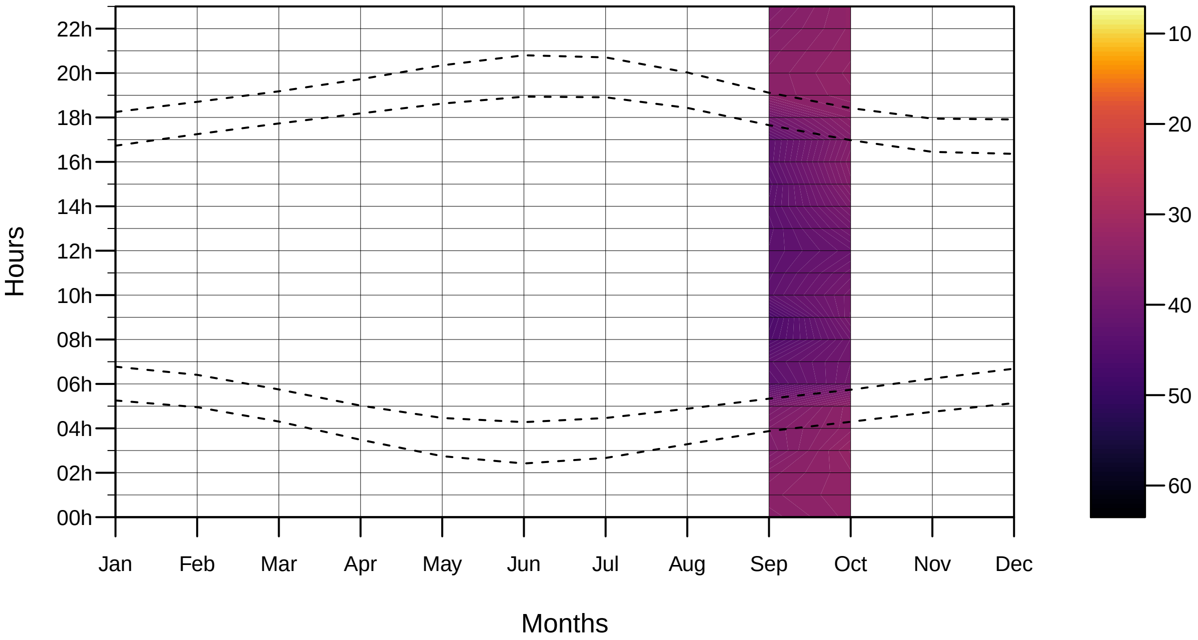

22h
20h
18h
16h
14h
12h
10h
08h
06h
04h
02h
00h
Hours
Jan
Feb
Mar
Apr
May
Jun
Jul
Aug
Sep
Oct
Nov
Dec
Months
10
20
30
40
50
60

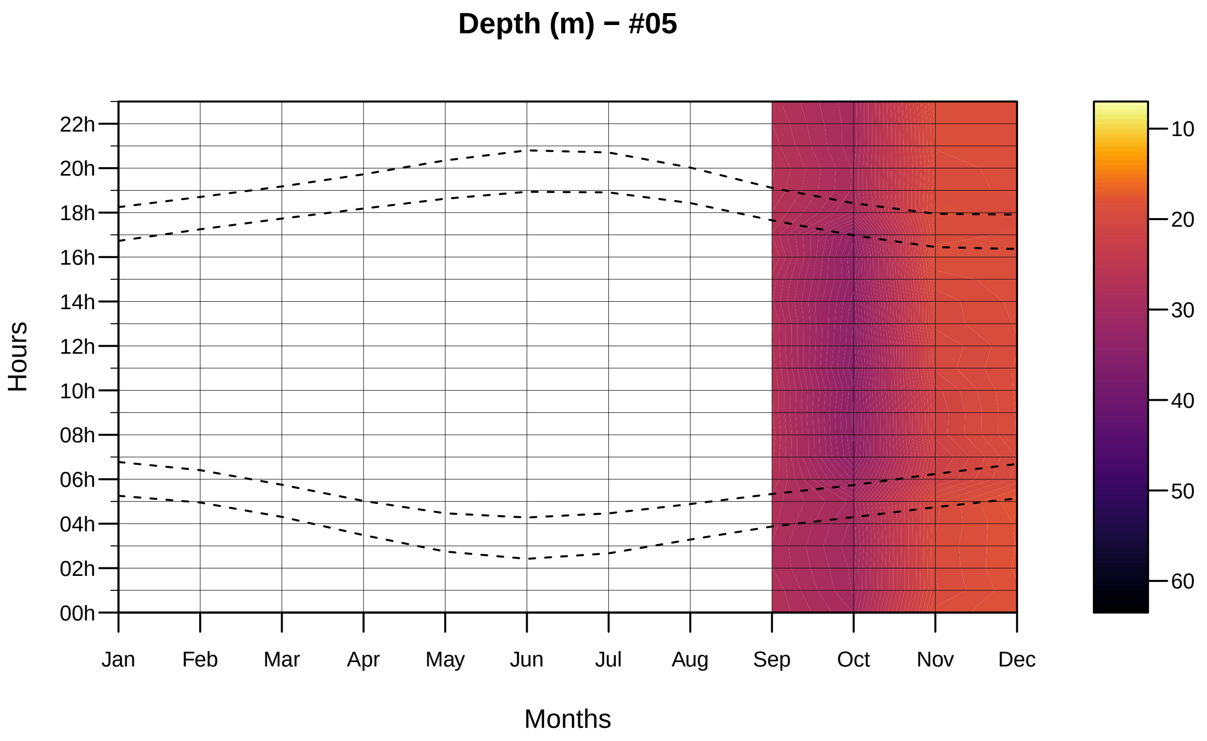

Depth (m) − #05
Hours
22h
20h
18h
16h
14h
12h
10h
08h
06h
04h
02h
00h
Jan
Feb
Mar
Apr
May
Jun
Jul
Aug
Sep
Oct
Nov
Dec
Months
10
20
30
40
50
60

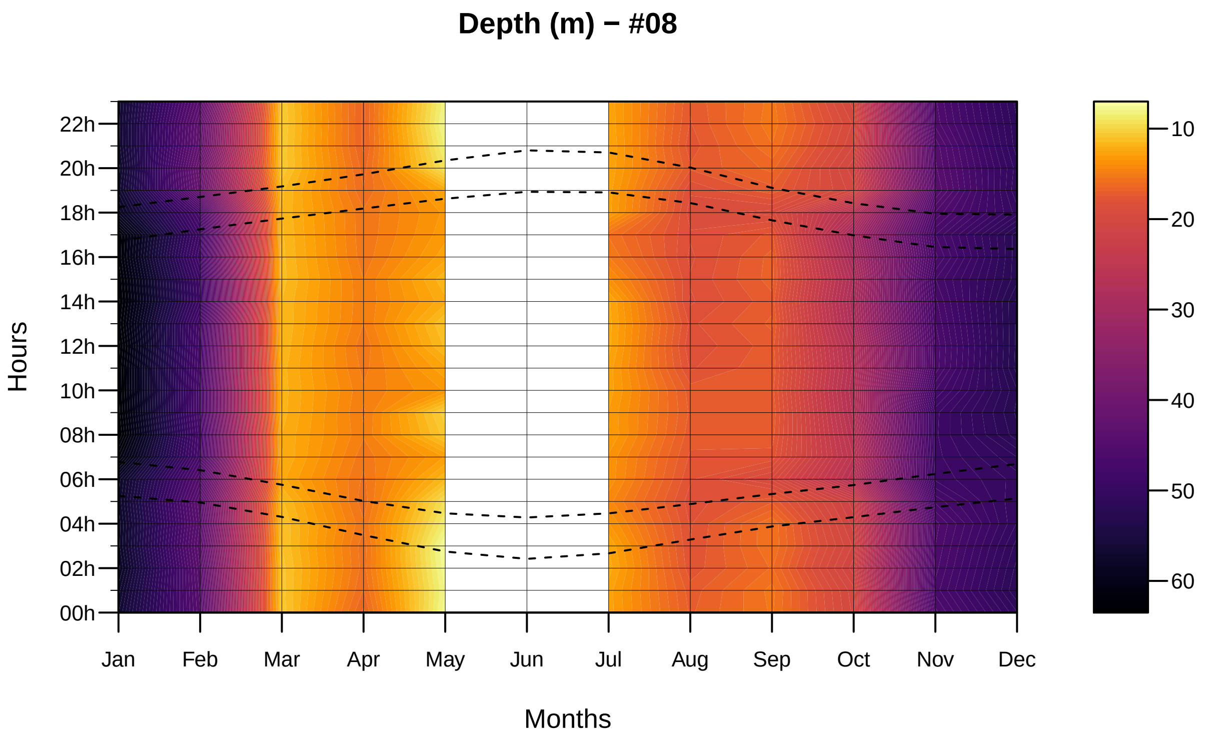
Depth (m) − #08
Hours
22h
20h
18h
16h
14h
12h
10h
08h
06h
04h
02h
00h
Jan
Feb
Mar
Apr
May
Jun
Jul
Aug
Sep
Oct
Nov
Dec
Months
10
20
30
40
50
60

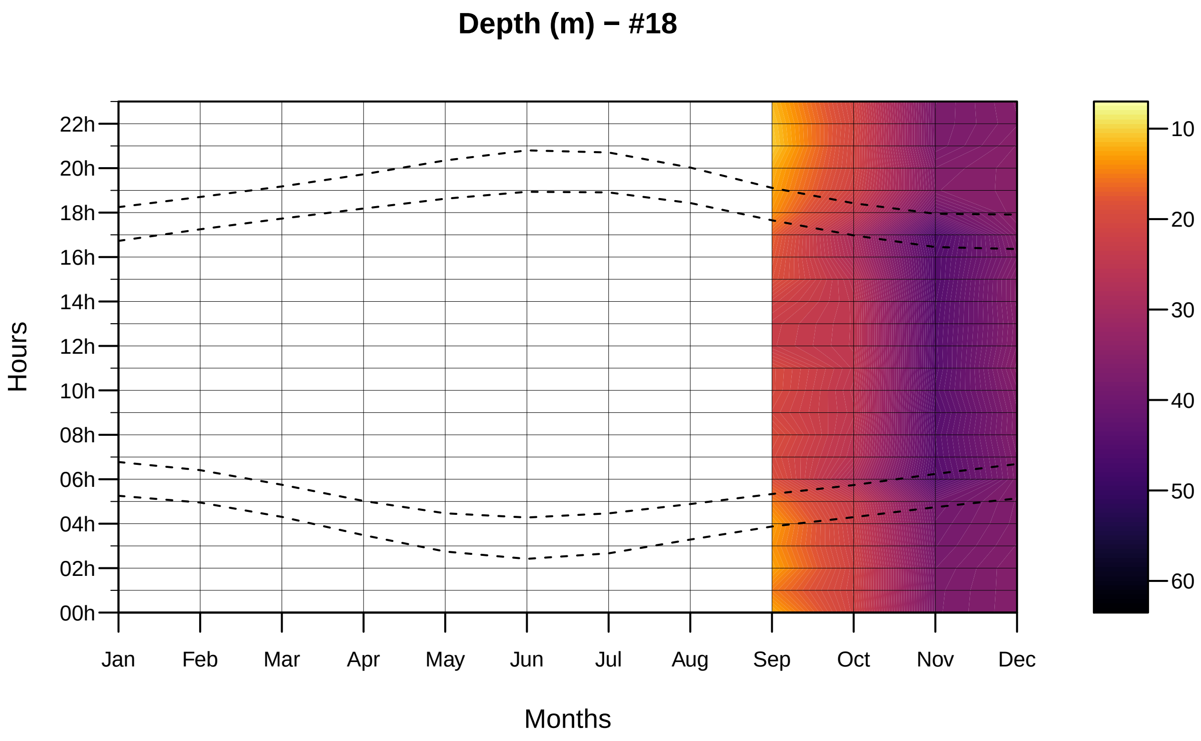
Depth (m) − #18
Hours
22h
20h
18h
16h
14h
12h
10h
08h
06h
04h
02h
00h
Jan
Feb
Mar
Apr
May
Jun
Jul
Aug
Sep
Oct
Nov
Dec
Months
10
20
30
40
50
60

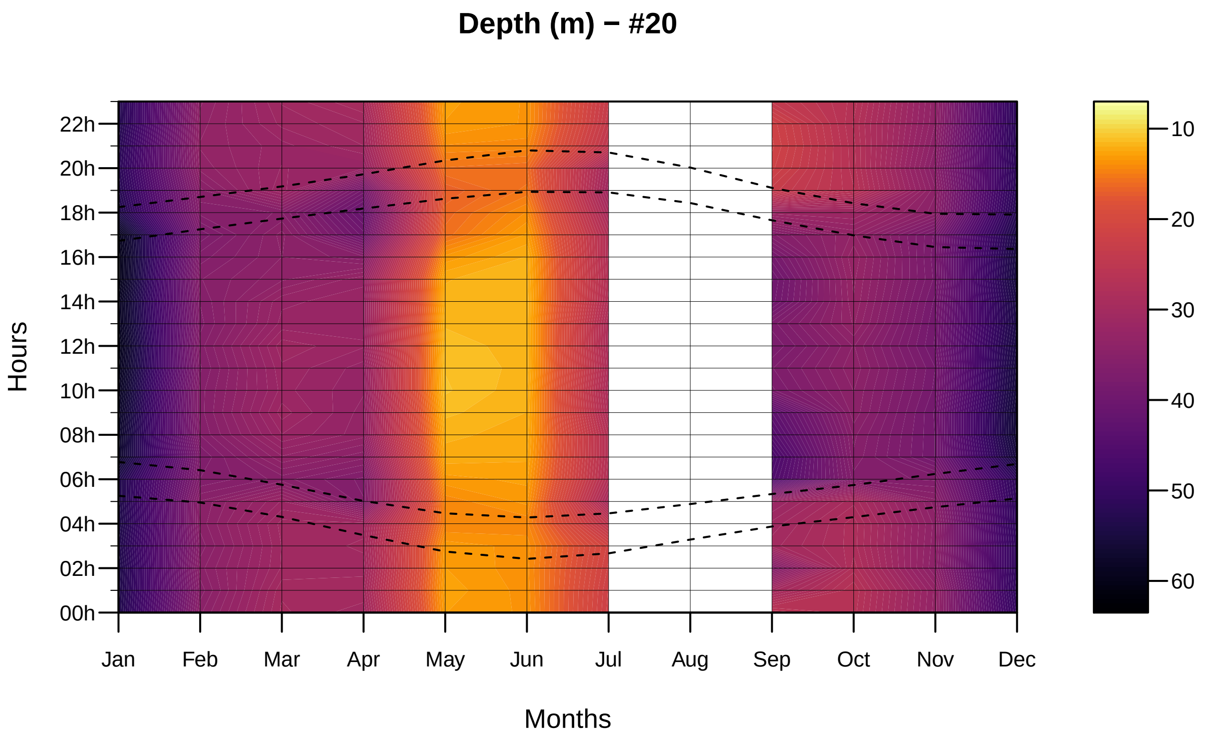

Depth (m) − #20
Hours
22h
20h
18h
16h
14h
12h
10h
08h
06h
04h
02h
00h
Jan
Feb
Mar
Apr
May
Jun
Jul
Aug
Sep
Oct
Nov
Dec
Months
10
20
30
40
50
60

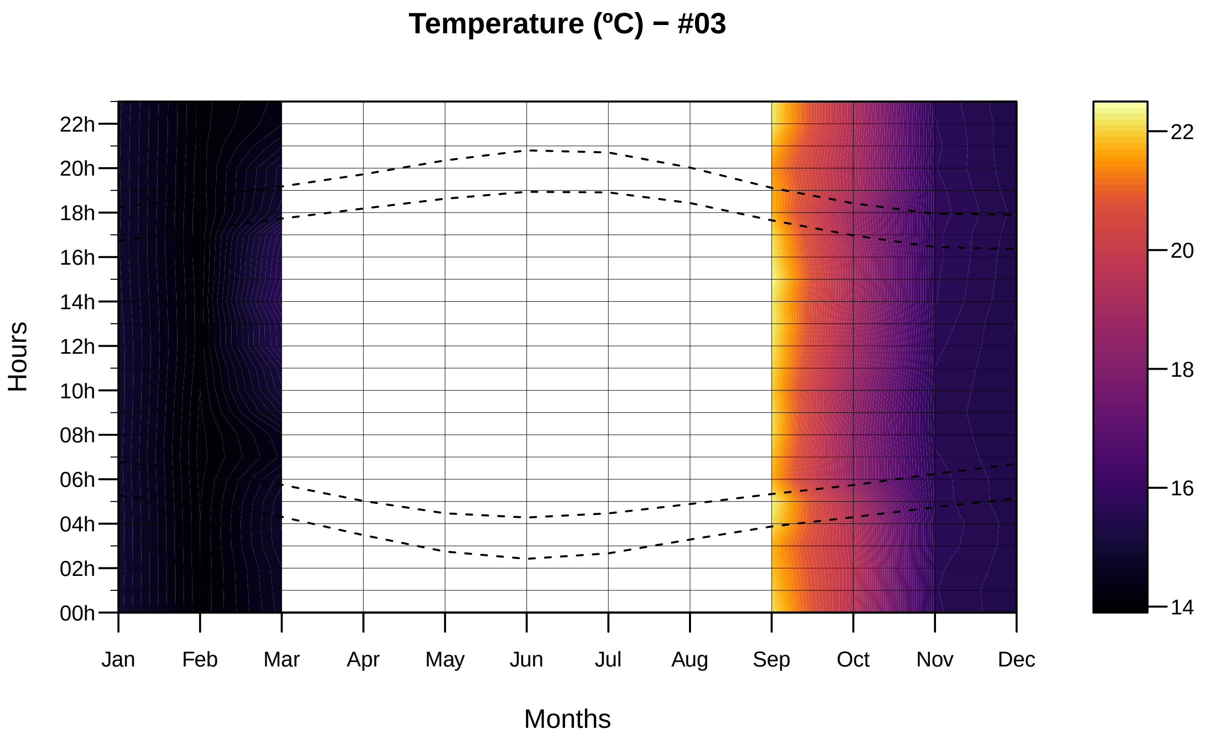
Temperature (ºC) − #03
Hours
22h
20h
18h
16h
14h
12h
10h
08h
06h
04h
02h
00h
Jan
Feb
Mar
Apr
May
Jun
Jul
Aug
Sep
Oct
Nov
Dec
Months
22
20
18
16
14

Temperature (ºC) − #04

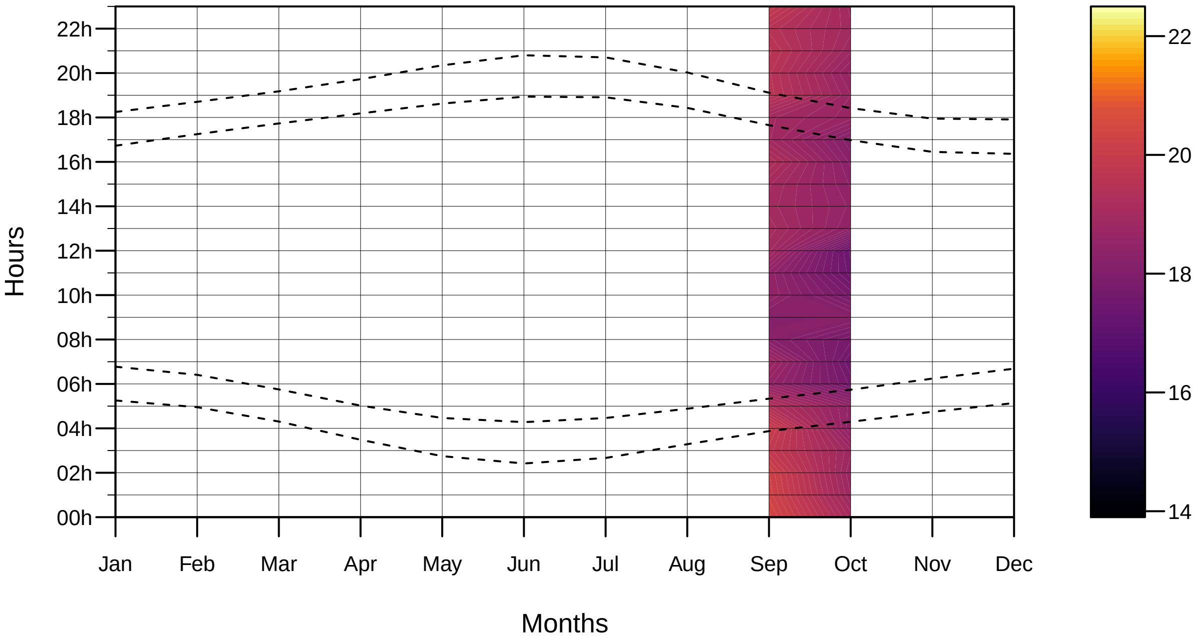

Temperature (ºC) − #05

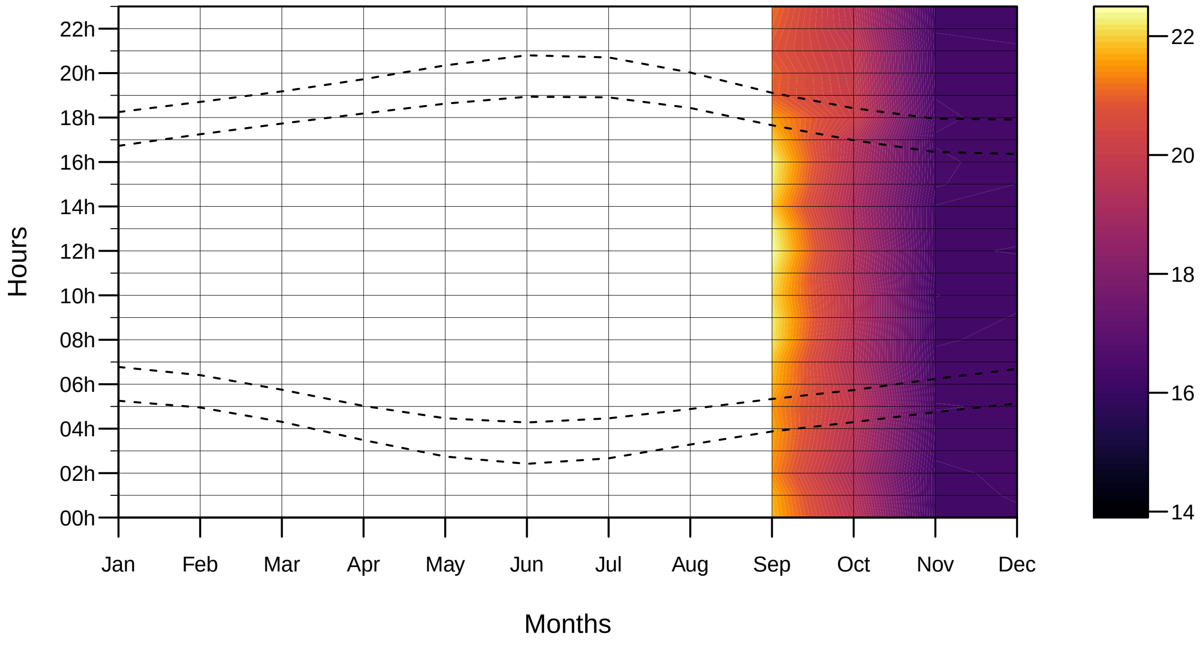

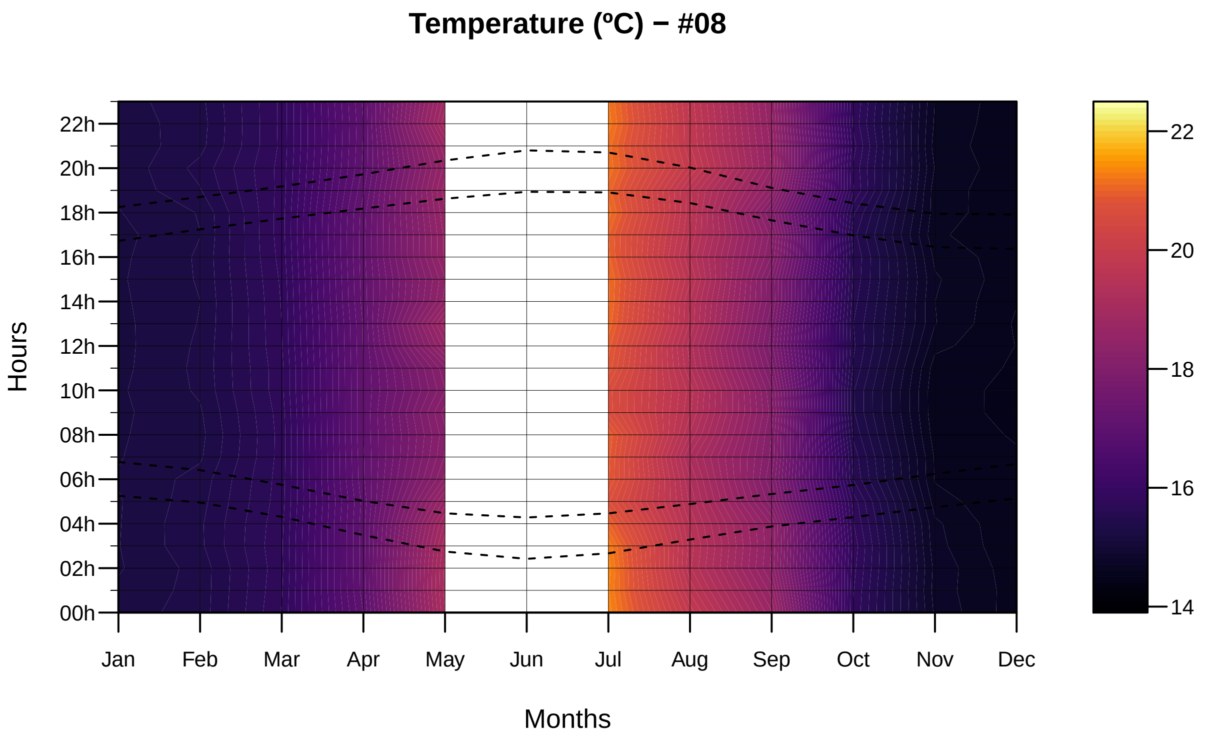

Temperature (ºC) − #08
Hours
22h
20h
18h
16h
14h
12h
10h
08h
06h
04h
02h
00h
Jan
Feb
Mar
Apr
May
Jun
Jul
Aug
Sep
Oct
Nov
Dec
Months
22
20
18
16
14

Temperature (ºC) − #18

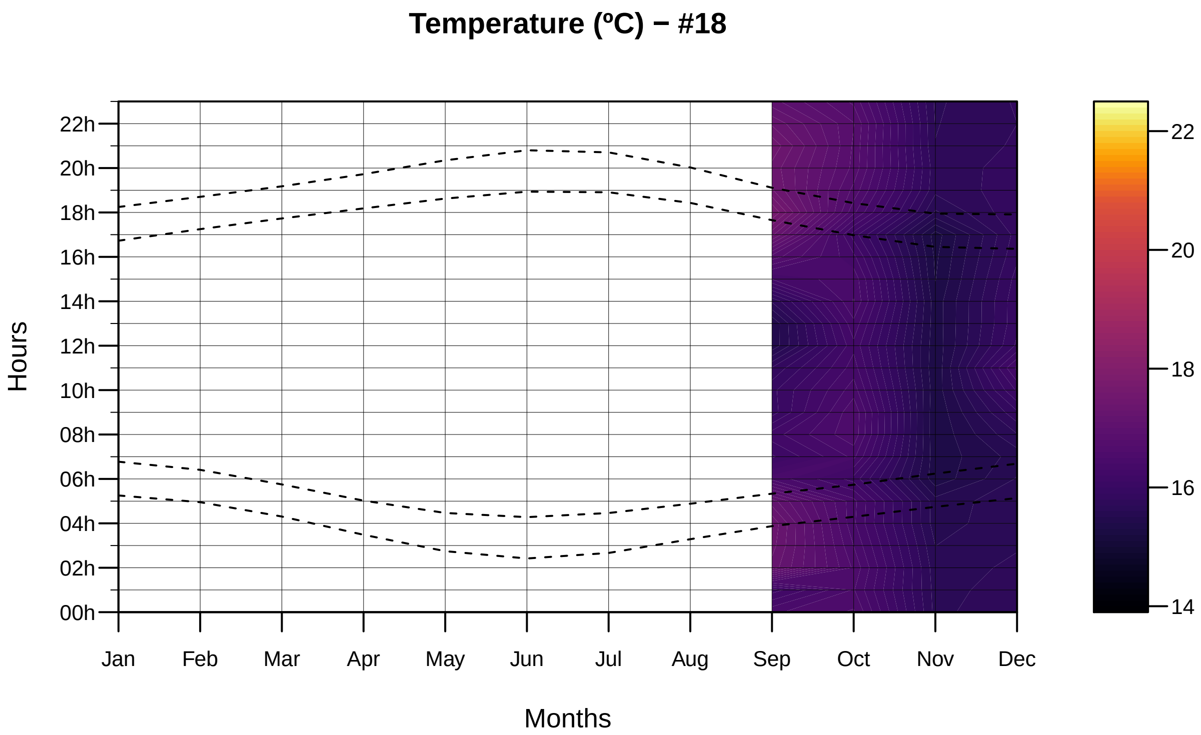

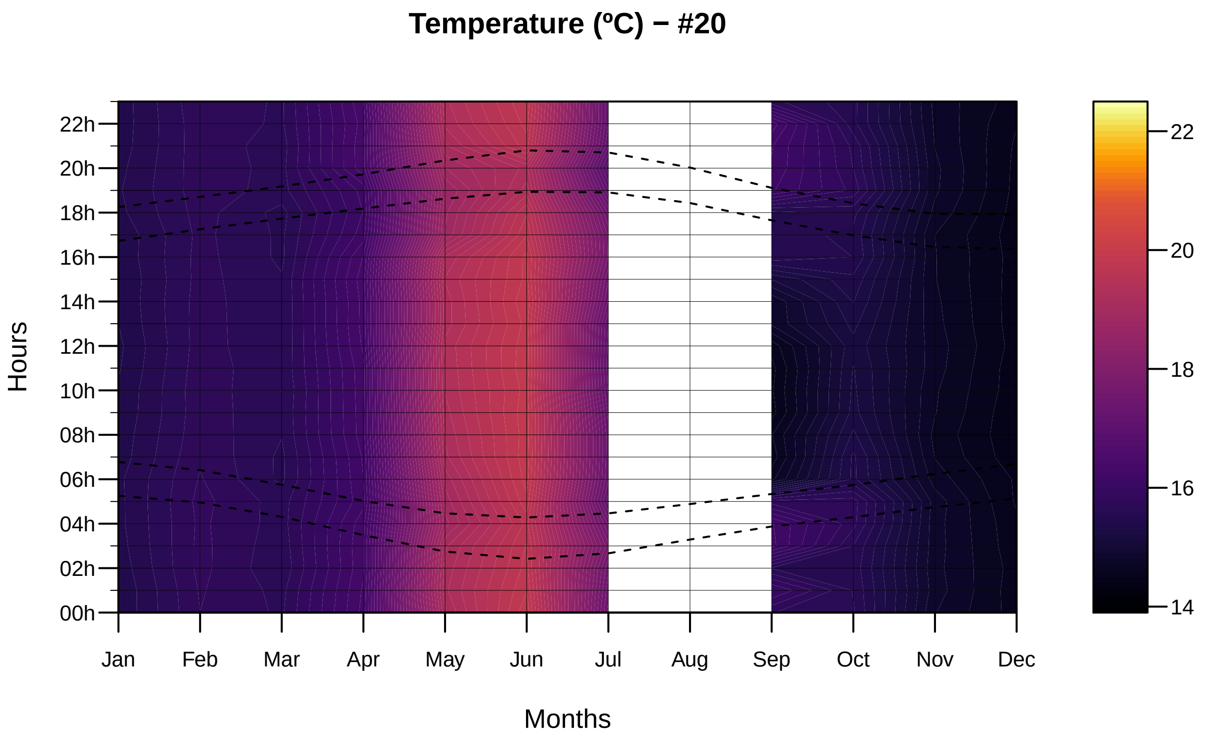

Temperature (ºC) − #20
Hours
22h
20h
18h
16h
14h
12h
10h
08h
06h
04h
02h
00h
Jan
Feb
Mar
Apr
May
Jun
Jul
Aug
Sep
Oct
Nov
Dec
Months
22
20
18
16
14
