# Supplementary material for: Depth and temperature preferences of meagre, Argyrosomus regius, as revealed by satellite telemetry
Source: PLoS One. 2023 Nov 21;18(11):e0288706. doi: 10.1371/journal.pone.0288706 (PMC10662727; doi:10.1371/journal.pone.0288706)
Supplement: S1 File — (DOCX) [file pone.0288706.s002.docx]

**Supplementary information**

**
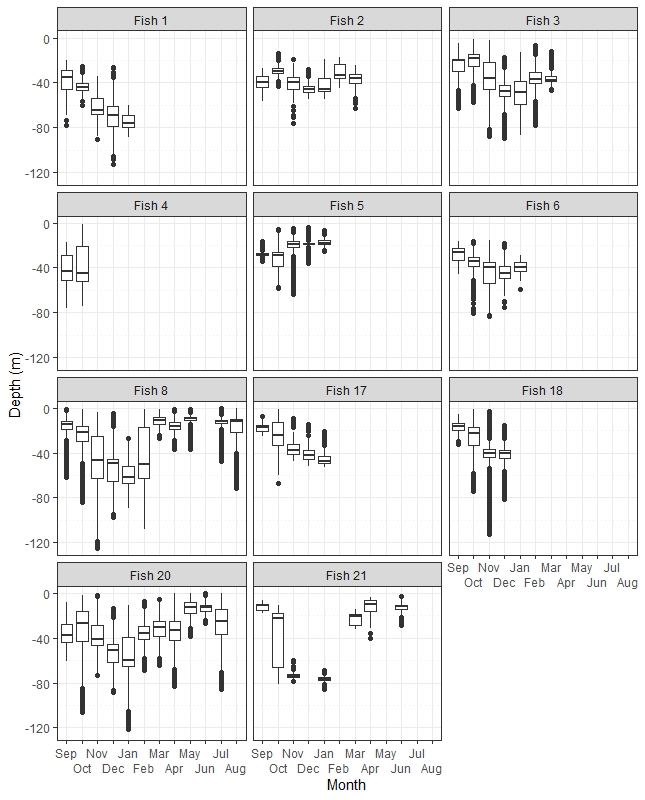
**

Figure S2. Monthly depth distribution recorded by adult *Argyrosomus regius* individuals tagged with PSAT archival tags in 2018 (Fish 1, Fish 2, Fish 3, Fish 4, Fish 5, Fish 6) and in 2019 (Fish 8, Fish 17, Fish 18, Fish 20, Fish 21).


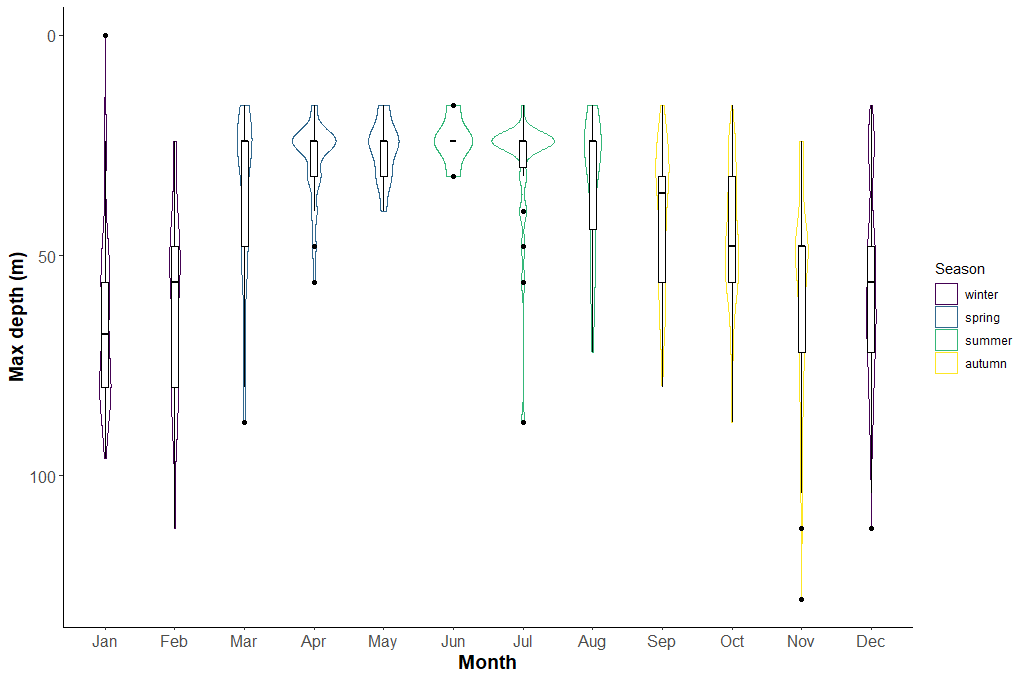


Figure S3. Monthly and seasonal maximum dive depth distribution recorded by all tagged adult *Argyrosomus regius* individuals tagged with PSAT archival tags in 2018 and in 2019.


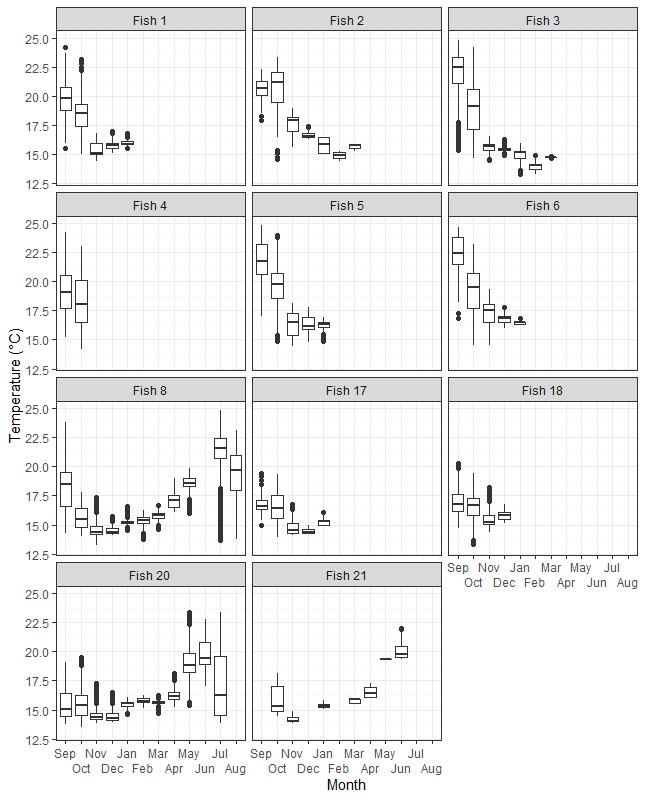


Figure S4. Monthly temperature distribution recorded by adult *Argyrosomus regius* individuals tagged with PSAT archival tags in 2018 (Fish 1, Fish 2, Fish 3, Fish 4, Fish 5, Fish 6) and in 2019 (Fish 8, Fish 17, Fish 18, Fish 20, Fish 21).

Table S1. Mean ± (standard error), maximum and the difference between mean day and night depth for all adult *Argyrosomus regius* individuals tagged with PSAT archival tags in 2018 (Fish 1, Fish 2, Fish 3, Fish 4, Fish 5, Fish 6) and in 2019 (Fish 8, Fish 17, Fish 18, Fish 20, Fish 21).

Table S2. Mean ± (standard error), maximum, minimum and the difference between mean day and night temperature for all adult *Argyrosomus regius* individuals tagged with PSAT archival tags in 2018 (Fish 1, Fish 2, Fish 3, Fish 4, Fish 5, Fish 6) and in 2019 (Fish 8, Fish 17, Fish 18, Fish 20, Fish 21).

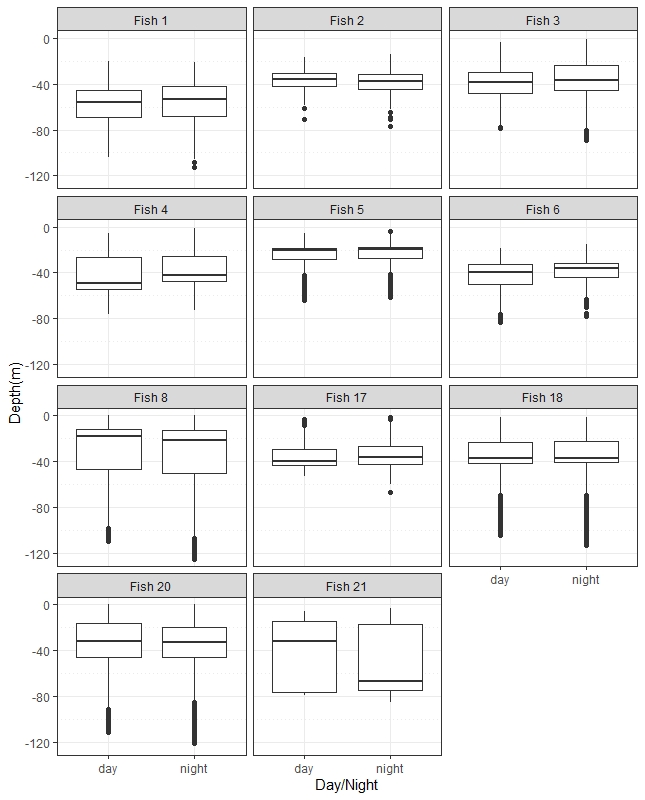


Figure S5 Day and night depth distribution recorded by adult *Argyrosomus regius* individuals tagged with PSAT archival tags in 2018 (Fish 1, Fish 2, Fish 3, Fish 4, Fish 5, Fish 6) and in 2019 (Fish 8, Fish 17, Fish 18, Fish 20, Fish 21).


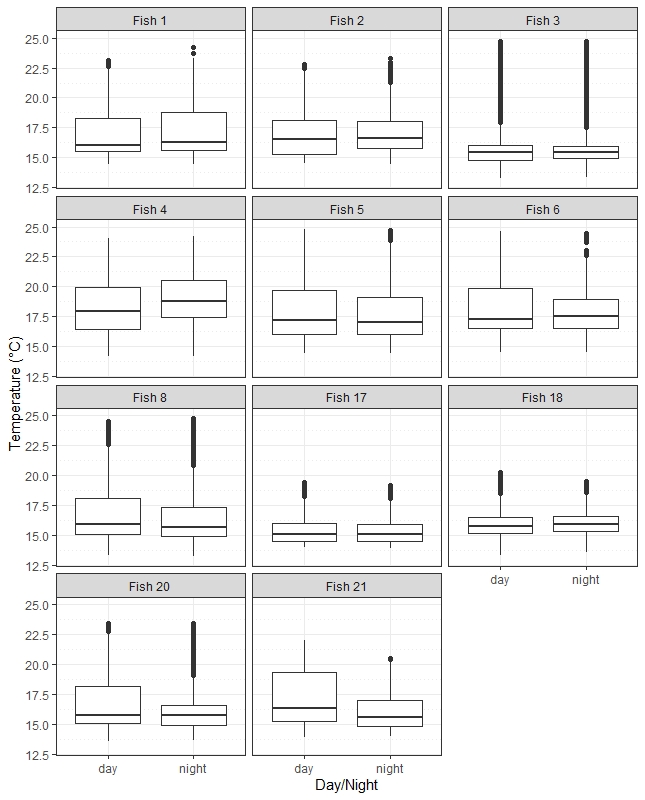
Figure S6 Day and night temperature distribution recorded by adult *Argyrosomus regius* individuals tagged with PSAT archival tags in 2018 (Fish 1, Fish 2, Fish 3, Fish 4, Fish 5, Fish 6) and in 2019 (Fish 8, Fish 17, Fish 18, Fish 20, Fish 21).


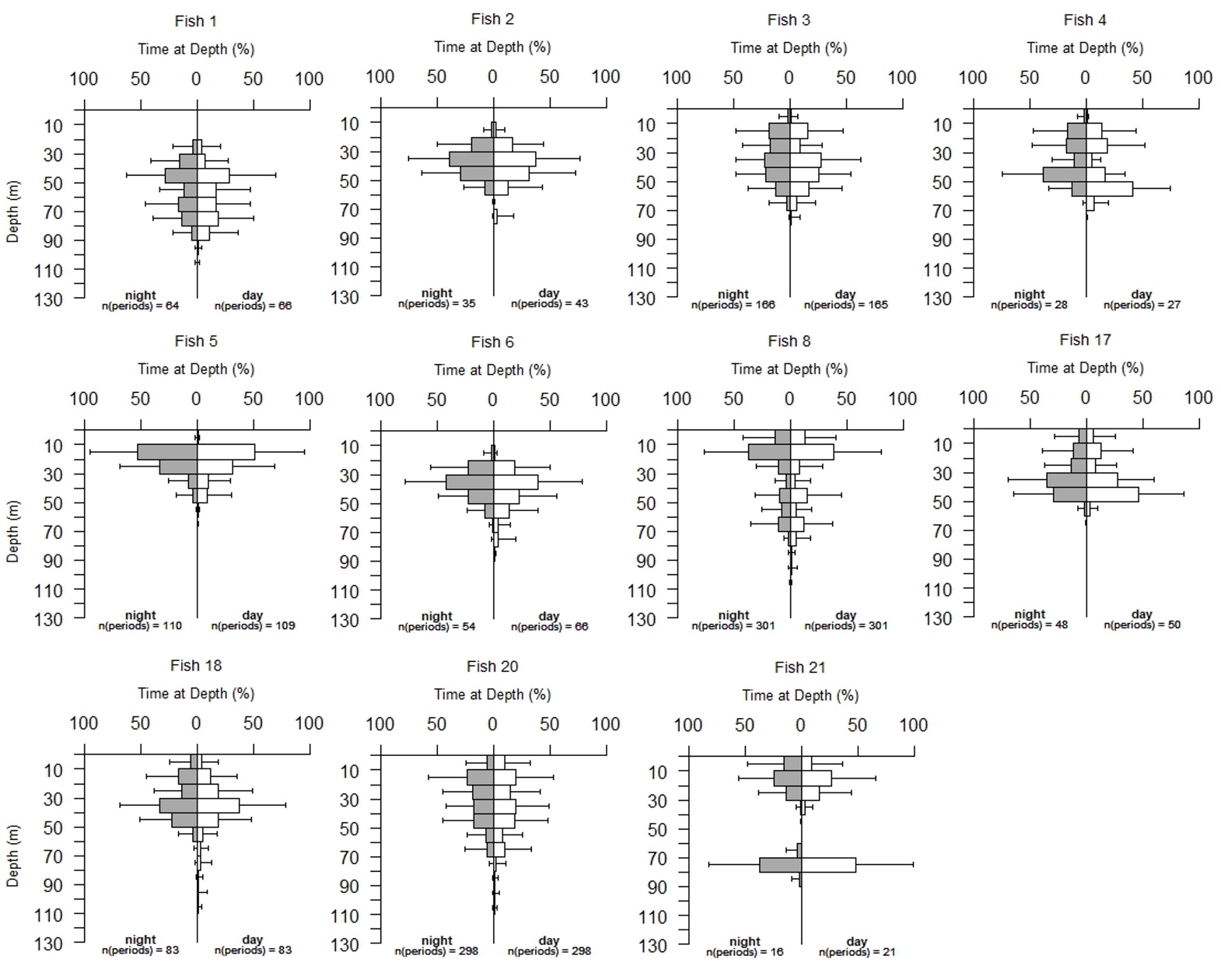


Figure S7. Time at depth diel histograms for adult *Argyrosomus regius* individuals tagged with PSAT archival tags in 2018 (Fish 1, Fish 2, Fish 3, Fish 4, Fish 5, Fish 6) and in 2019 (Fish 8, Fish 17, Fish 18, Fish 20, Fish 21).


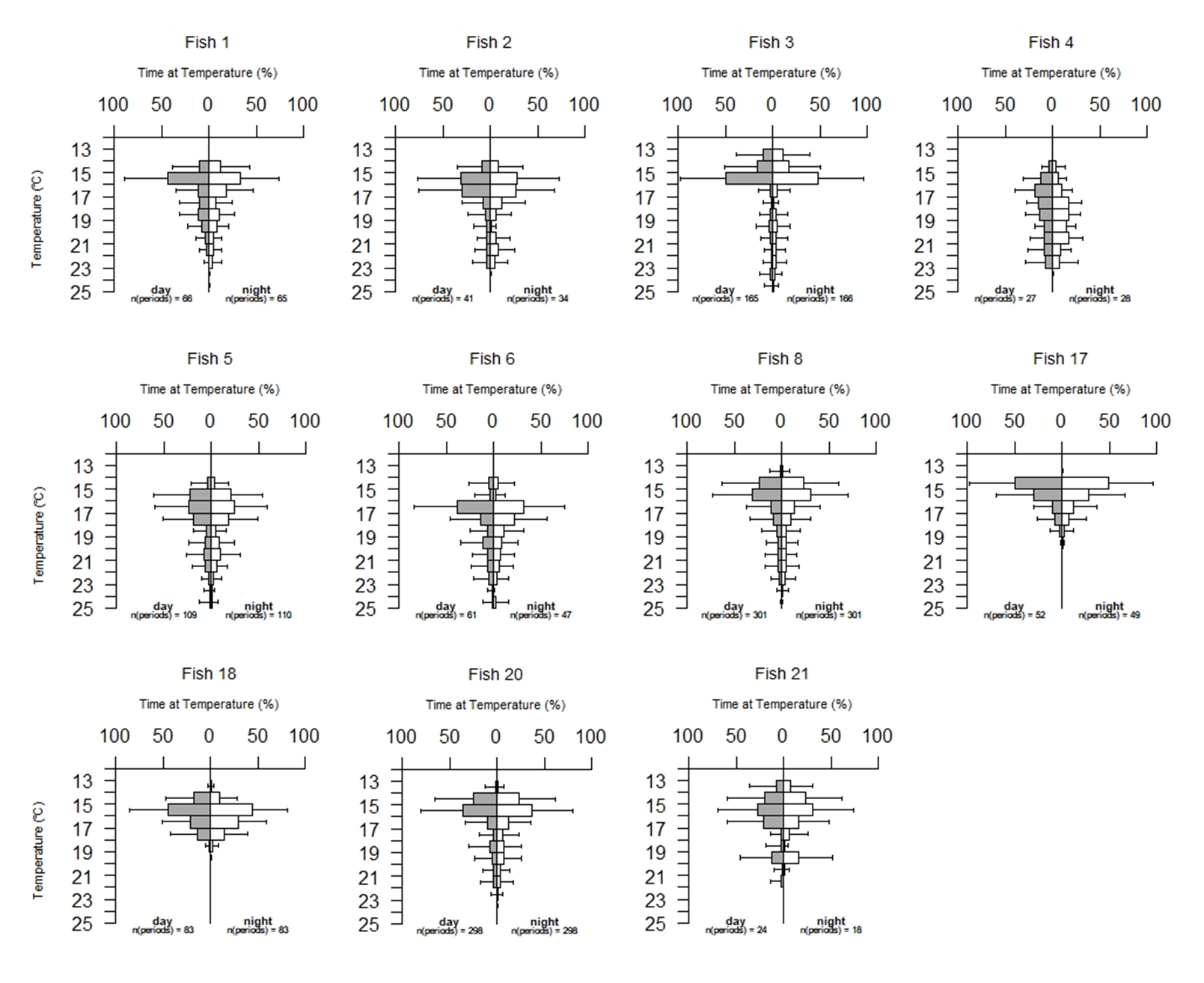


Figure S8. Time at temperature diel histograms for adult *Argyrosomus regius* individuals tagged with PSAT archival tags in 2018 (Fish 1, Fish 2, Fish 3, Fish 4, Fish 5, Fish 6) and in 2019 (Fish 8, Fish 17, Fish 18, Fish 20, Fish 21).
