# Supplementary figures and images for: Depth and temperature preferences of meagre, Argyrosomus regius, as revealed by satellite telemetry
Source: PLoS One. 2023 Nov 21;18(11):e0288706. doi: 10.1371/journal.pone.0288706 (PMC10662727; doi:10.1371/journal.pone.0288706)

## Depth (m) – #03

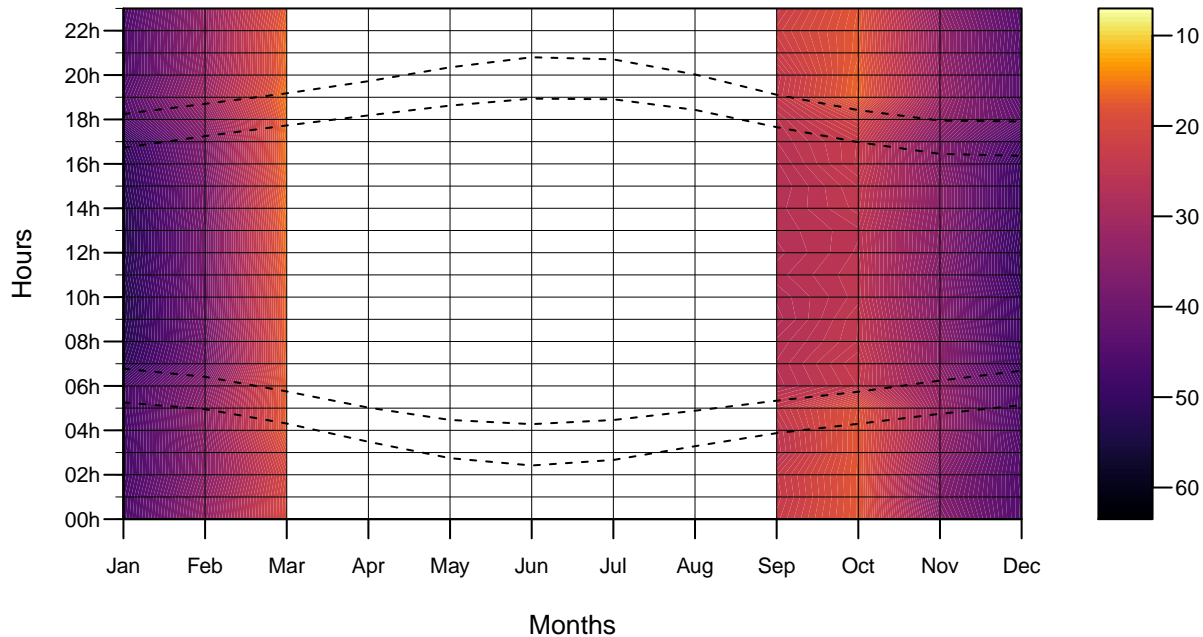

## Depth (m) – #04

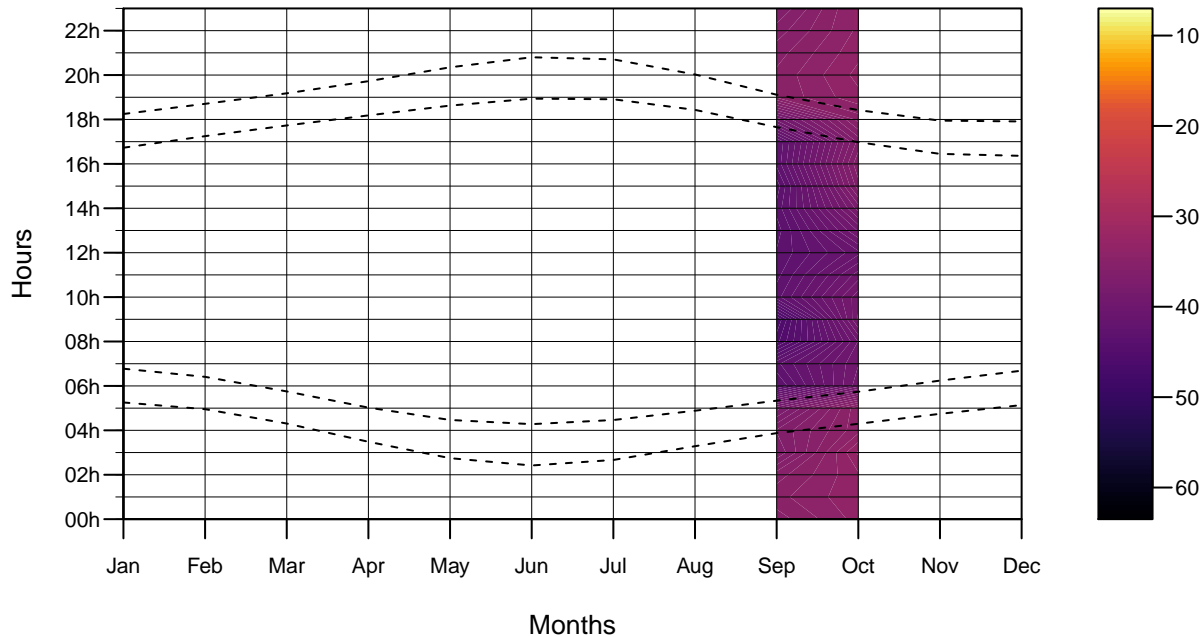

### Depth (m) – #05

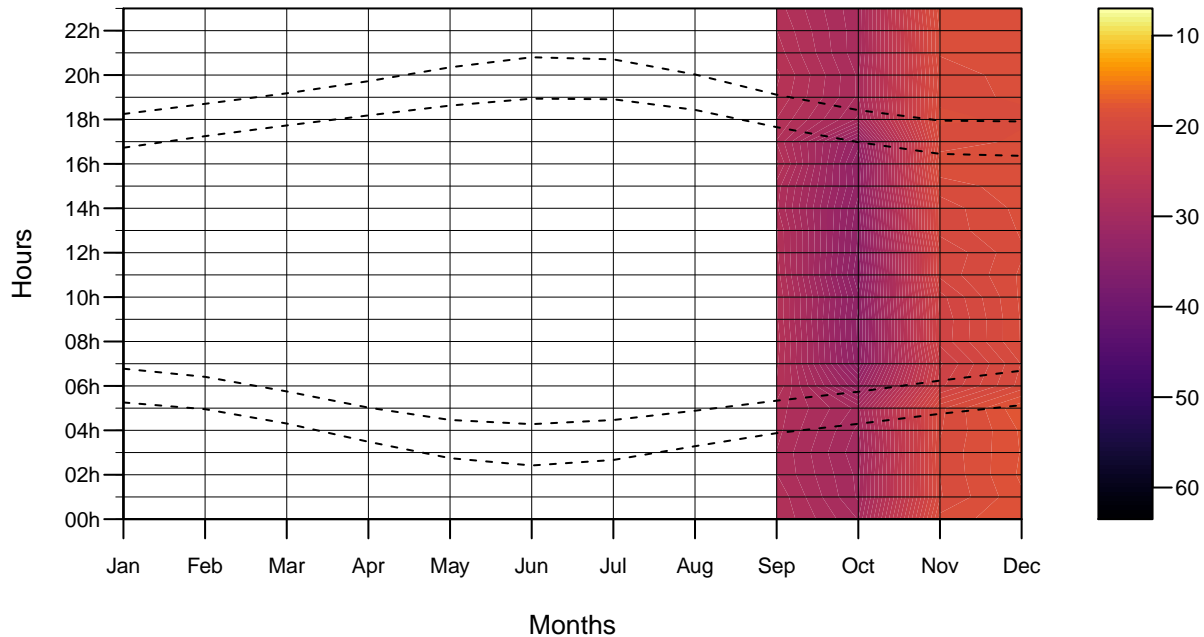



### Depth (m) – #18

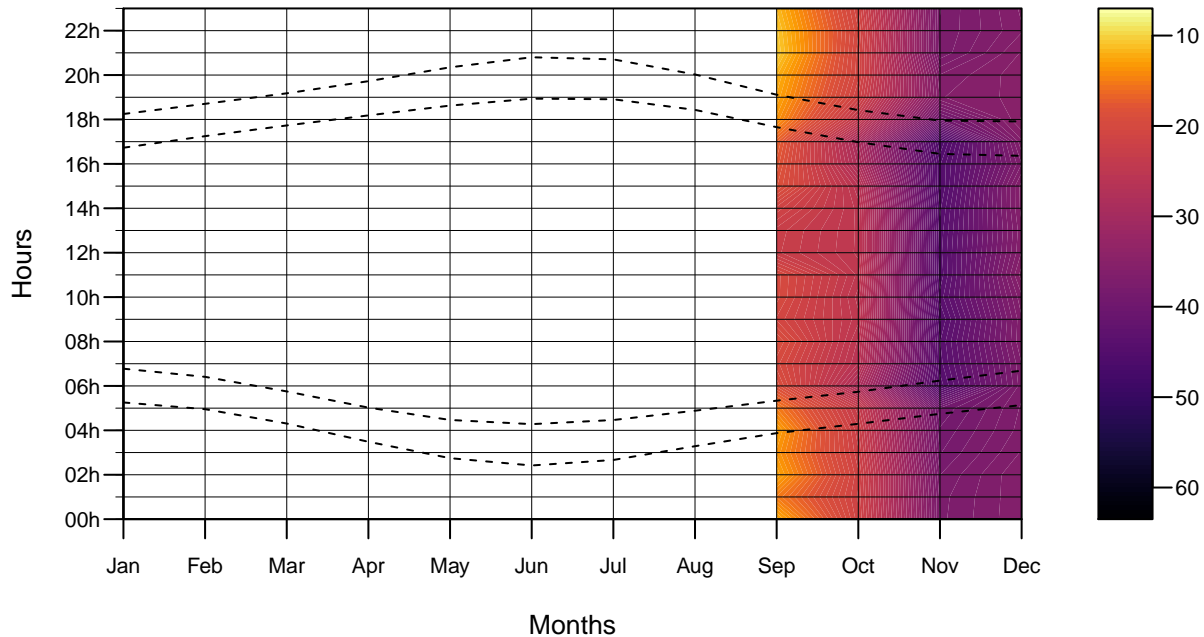

Supplement: S1 Data — (PDF) [file pone.0288706.s003.pdf]

# Temperature (°C) – #03

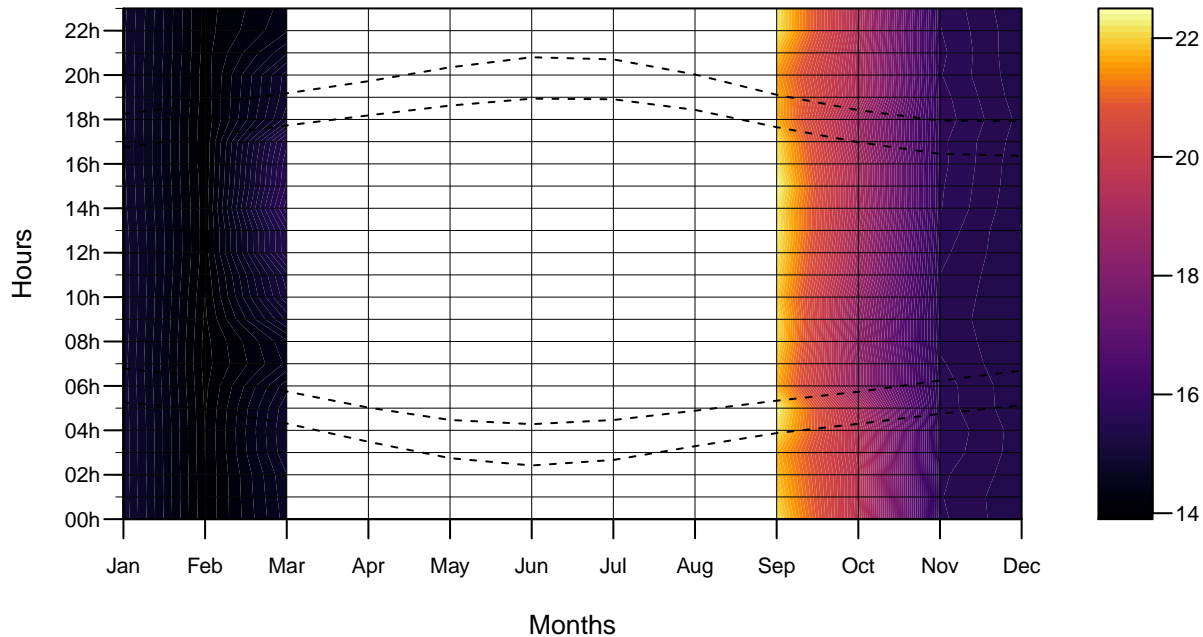

## Temperature (°C) – #04

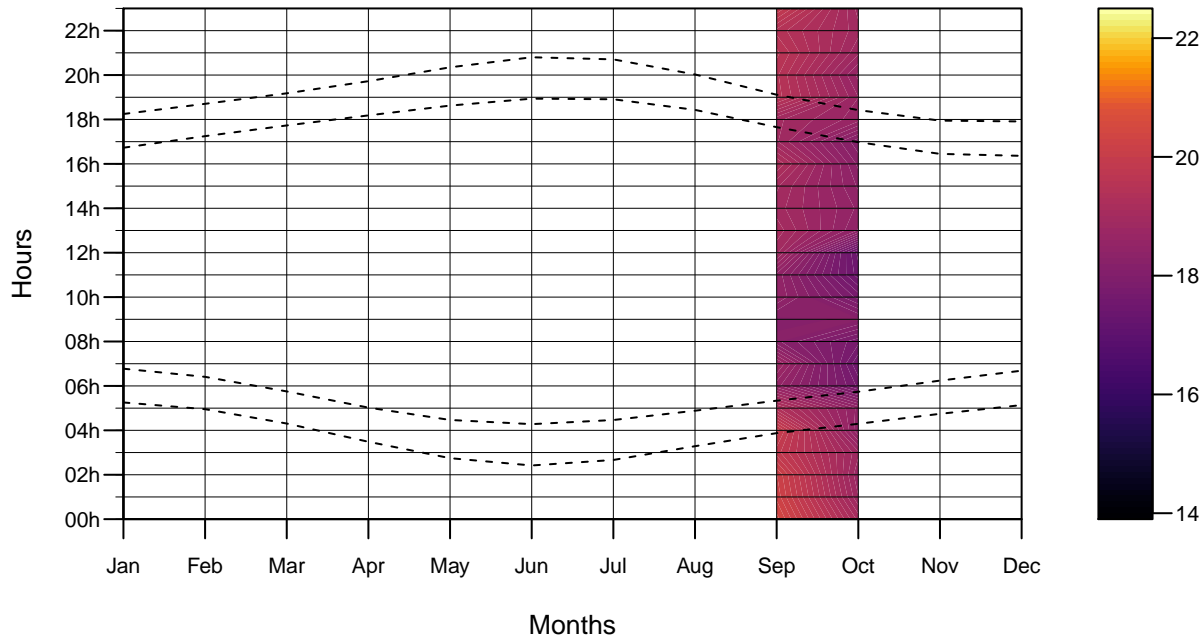

## Temperature (°C) – #05

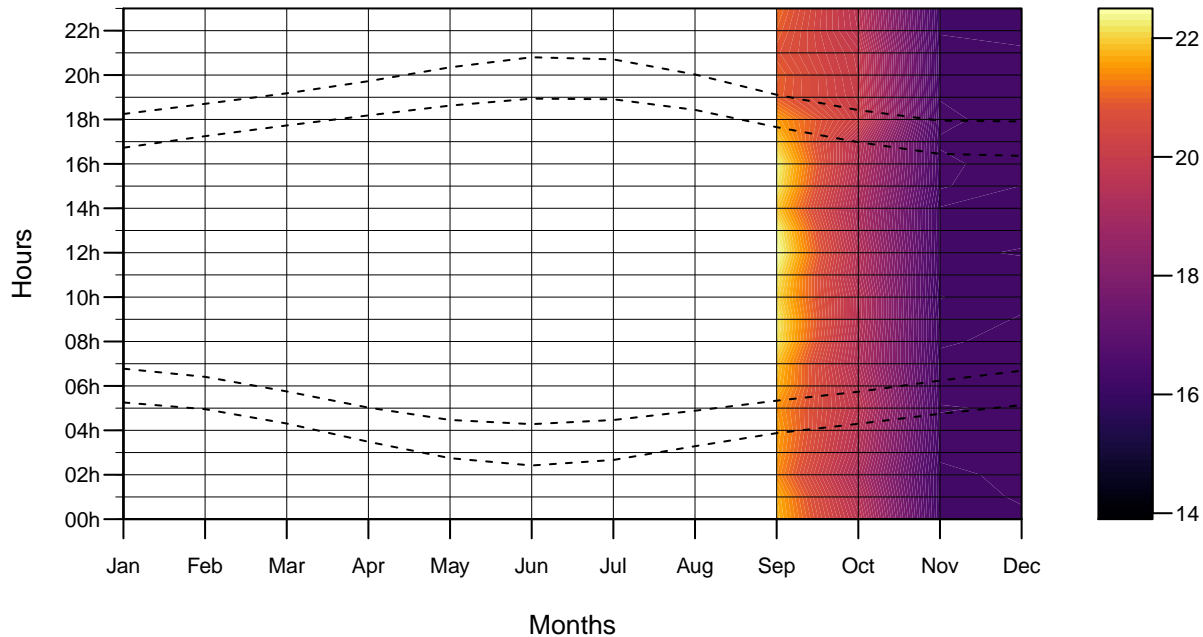



### Temperature (°C) – #18

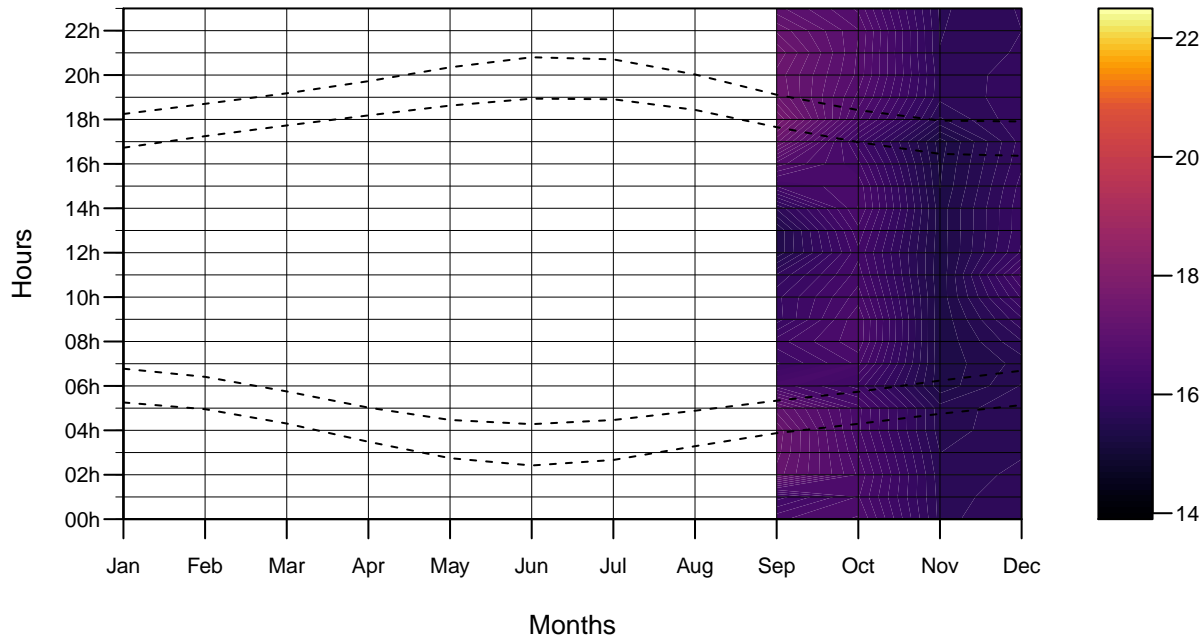

## Temperature (°C) – #20

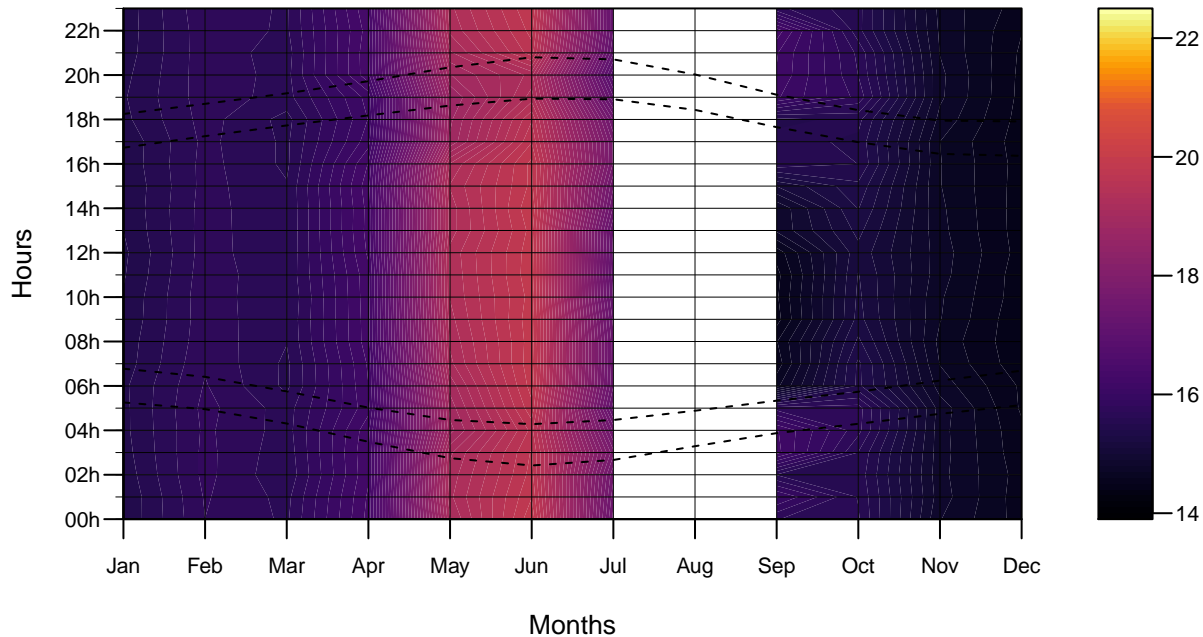

Supplement: S2 Data — (PDF) [file pone.0288706.s004.pdf]
