# Supplementary material for: Depth and temperature preferences of meagre, Argyrosomus regius, as revealed by satellite telemetry
Source: PLoS One. 2023 Nov 21;18(11):e0288706. doi: 10.1371/journal.pone.0288706 (PMC10662727; doi:10.1371/journal.pone.0288706)
Supplement: Attachment — Submitted filename: Response to Editor .docx [file pone.0288706.s005.docx]

**Dear Editor,**

**Again, we would like to thank you for accepting the manuscript for review in Plosone and appreciate the timely response from you and the reviewer. We too think that both your recommendation and those of the reviewer greatly benefit the manuscript. We have place particular care in the recommendation suggest adding extra detail to the analyses.**

**To make the re-evaluation process easier for yourself I have in this document responded directly to your critiques and suggestions which are highlighted in bold following your suggestions. All line numbers are referring to the document without track changes while a separate track changed document has too been uploaded.**

**I have added a large supplementary document which has additional figures and tables that have been asked for by both yourself and the reviewer. Our feeling however is that none of these additional figures and tables change the manuscripts outcomes and findings which is why I have refrained from including them in the main document. If you or the reviewer do feel as though that that they are needed to be added to the main document to back up our original finding I am happy to do so but if anything, I feel as though they simply back up what has already been presented.**

**I hope that you find our responses and corrections satisfactory so that you are able to consider the manuscript for publication.**

**Regards,**

**Authors**

Editor:

Dear Dr. Claus Winkler,

I would like to apologies for the long delay. One reviewer told me that he could send a report but I have not received any news for a few days. As such, in order to avoid any further delay, I decided to serve as a reviewer and take a decision using my opinion and the report of the reviewer who sent a report.
I think the dataset is interesting and provides a real addition to the literature as the behaviour of this species is poorly known. However, I tend to agree with the reviewer that some analyses need some considerations (i.e. GAMM) and that some additional analyses could be also conducted or other details provided (e.g. depth and temperature histograms). Another approach could be the use of Continuous wavelet transformations (CWTs) or FFTs to investigate for cyclical patterns in depth use.
Like the reviewer, I do not understand why depth is found as the response variable (i.e. in depth bin) and in the explanatory variable... this need to be addressed or explained.

**Author:**

**Again, we would like to thank you for accepting the manuscript for review in Plosone and appreciate the timely response from you and the reviewer. We too think that both your recommendation and those of the reviewer greatly benefit the manuscript. We have place particular care in the recommendation suggest adding extra detail to the analyses.**

**We acknowledge and understand the suggestion of using a GAMM for the modelling procedure for the manuscript and this was initially investigated but unfortunately due to a high level of overdispersion in the response variable we could not find an appropriate response variable data structure family to suit this data in any of the R packages that run GAMMs. In response to this issue that we faced it was decided to use a GAM in the MGCV R package that allows for the use of a quasibinomial response variable structure to cater for the data’s overdispersion. Due to the inherent dependence of telemetry data it was important to add fish ID as a random effect which I too did not think was possible in a GAM but through extended research into the top it was possible and there for included in the same way that is used in a GAMM or GLMM. Based on this we still think that the use of a GAM is appropriate for our data set as if we used a GAMM it would be harder to cater for the overdispersion of the data. While we agree that conventionally GAMMs have been used more to analyse telemetry data but there has been growing awareness of the flexibility of using GAM’s in the MGCV package to analyses telemetry data. This was recently highlighted in a recent telemetry work shop hosted by ETN (**[**https://europeantrackingnetwork.org/en/training-school-calculating-positions-aquatic-telemetry**](https://europeantrackingnetwork.org/en/training-school-calculating-positions-aquatic-telemetry)**) which had two days of workshops dedicated to using GAMs on telemetry data.**

**In addition to this we have included both temperature and depth distribution histograms for each fish and split by day and night as well as box plots of temperature and depth for both day and night asw well as by month. These are included in the manuscript as an appendix, but I am more than happy to add them as tables to main paper if needed but I do not think this is necessary. While we acknowledge the suggestion of using Continuous wavelet transformations (CWTs) or FFTs to assess cyclical patterns in the data we have added two tables as well as the boxplots that we think explore the potential diel effects on temperature and depth use.**

**As you will see from the tables and box plots the differences in the mean temperature and depth use during the day and night are very small ranging between a 5.7 m – 1.1 difference in mean depth between day and night. Based on the suggestions by the reviewer we too explored the option of using both parametric or non-parametric T-tests to explore whether there was a statistically significant differences between both depth and temperature between day and night. Unsurprisingly the data did not conform with assumptions of a T-test so we conducted Two-sample Kolmogorov-Smirnov (K-S) as suggested by the reviewer, unsurprisingly given the size of the datasets and the very small standard errors significant effects of day and night on depth and temperature use were found for all fish (P< 0.05). We, however, do not think this is an appropriate way of analysing this data given the size of this massive dataset per individual essentially those individuals from which tags were recovered. Given the size of the dataset and based on the size of the standard errors (very small) on most occasion if a diel difference in mean depth corresponds to a difference one or two meters these tests will find a significant effect. The question is whether this is biologically relevant in this study? At the great mean diel differences in depth and temperature of 5.7 m and 0.9 ˚C respectively of a fish that utilises a depth range of 0 – 125 m and fish that are over 1 m in length we do not think that the results of these tests are anything to go by. Please read the answer to the blog regarding the use of such analyses on large data sets where it is stated that: “With a large enough sample size, the hypothesis test can detect an effect that is so minuscule that it is meaningless in a practical sense” (https://statisticsbyjim.com/hypothesis-testing/practical-statistical-significance/)**

**The reason we did not include day and night into the GAM was due to use exploring daily explanatory variables and by introducing day and night into the dataset we would essentially be doubling the N numbers causing the model not to converge which is why we stuck to daily data.**

**I apologise regarding the confusion around the response variable, but time spent within each depth bin was the proportional (binomial) response variable while each depth bin was recoded as continuous and used as an explanatory variable see: https://stat.ethz.ch/pipermail/r-help/2010-March/232580.html.**
Here are additional comments:

Line 56: present only in the Eastern Mediterranean?

**Line 57: Rectified to the entire Mediterranean, “eastern” ommitted**

Line 208-209: I do not understand the difference between ‘programmed period’ and ‘entire time planned’...

**Line 218 -219: Changed from Programmed period to “programmed deployment” and “entire time planned changed to “programmed deployment”.**

Line 213: check the spaces between the numbers ‘261 121 81’

**Line 223: Apologise changed to: 26 121 181**

Figure 1: It could have been nice, if available, to add a picture of these traps and a picture of a tagged meagre (from Figure S1 for example)

**The Figure has been changed with pictures of the trap and an insert of a tagged *A. regius***

As such, I invite you to submit a revised version of your manuscript addressing the reviewer’ comments and additional ones I provided above.

Kind regards

Johann


[Note: HTML markup is below. Please do not edit.]

Reviewers' comments:

Reviewer's Responses to Questions
