# Supplementary material for: Depth and temperature preferences of meagre, Argyrosomus regius, as revealed by satellite telemetry
Source: PLoS One. 2023 Nov 21;18(11):e0288706. doi: 10.1371/journal.pone.0288706 (PMC10662727; doi:10.1371/journal.pone.0288706)
Supplement: Attachment — Submitted filename: Response to reviewer.docx [file pone.0288706.s006.docx]

Dear reviewer,

In response to your last comment:

” I do, however, remain unconvinced about the inclusion of depth as a smoother in the model. What purpose was this serving? It still seems to me that the response variable is not independant from this explanatory variable (high time in a certain depth bin will inherently be highly correlated with the depth smoother).”

Firstly, we would like to thoroughly apologise for the delay in the resubmission of this manuscript, we acknowledged your concerns regarding the statistical modelling approach used. We agree that the approach initially used had some severe statistical flaws and acknowledge that the response variable and the depth explanatory variable were depended on each other. Unfortunately, given this hurdle we spent a large amount of time debating the right approach. We therefore decided to remove this analysis and use a simpler statistical approach using a Kruskal-Wallis rank sum test after investigating the assumptions equal variance, to compare the maximum depths per month purely based on individual fish maximum diving depth. This was followed by a post-hoc Games-Howell test, to identify statistical significance amongst months. Fortunately, this different approach did not affect the main findings of the manuscript, so we simply replaced the old analysis in the methods and the results with the new simpler analysis. The new figure, post-hoc analysis table and an associated supplementary figure have been added to the manuscript, the previous analysis and associated table have been removed.

We hope that the omission of the old analysis and the inclusion of the new analysis both satisfies recommendations. We look forward to your response and thoroughly apologise for the delay in the resubmission of the manuscript

On behalf of all the authors,
