# Supplementary material for: Depth and temperature preferences of meagre, Argyrosomus regius, as revealed by satellite telemetry
Source: PLoS One. 2023 Nov 21;18(11):e0288706. doi: 10.1371/journal.pone.0288706 (PMC10662727; doi:10.1371/journal.pone.0288706)
Supplement: Attachment — Submitted filename: reviewer comments addressed.docx [file pone.0288706.s007.docx]

Dear Reviewer,

We would like to thank you for your comments and suggestions which we feel as though greatly improve the manuscript. We have spent considerable effort in trying to respond to your suggestions which have been specifically outlined below, please refer to the line numbers in the version of the manuscript which lacks track changes. We hope that we have adequality addressed all your comments and hope that the manuscript maybe considered for publication in PlosOne. Our responses to the your comments are highlighted in bold for easier following.

Regards,

Authors,

Specific comments

L16 The combination of “demand for this species, and their swim bladders” sounds a bit strange in the abstract without further explanation. Consider removing or rephrasing (e.g., demand for this species, particularly their swim bladders; demand for this species flesh and, in most recent times, swim bladders for the Asian market…).

**See line 16: We have followed the reviewer’s suggestion. This sentence now reads “Demand for this species, and more recently for their swim bladders, has led to regional population declines and growing importance as an aquaculture species”**

L25 do not venture

**See line 25: Changed accordingly.**

L46 consider: “thermal preferences may be used as one of the main factors to explain” (i.e., it is true temperature explains a lot but there are other factors that condition species distribution- prey abundance, dissolved oxygen..).

**See lines 43-46: We rephrased this sentence. It now reads “Within these complexities and environmental interdependencies of the habitat selection, temperature is known to be a strong determinant of fish distribution, meaning that thermal preferences may be one of the main factors to explain and predict a species’ response to changes in oceanographic conditions related to weather, season, or climate [8, 9].”**

L52 Consider the following change: The depth and thermal envelopes occupied by a given species also define their environmental niche… (it is somehow obvious the depth envelope defines the vertical niche, as it is now).

**See lines 50-52: We agree with this comment and made changes accordingly. The sentence now reads “The depth and thermal envelopes occupied by a given species also define their environmental niche and, therefore, the organisms they can predate on, be predated by, or compete with.”**

L96 Additionally, in the case of A. regius (the previous sentences seem to be related to sciaenids in general)

**See line 96-98: We agree with this comment and made changes accordingly. The sentence now reads:” Additionally, in the case of *A*. *regius* these appear to be limited to just six areas within large estuary and deltas:”**

L118 The figure caption is embedded in the main text. Figure captions are generally placed elsewhere separately.

**Based on our understanding from the Plosone submission guidelines figure captions are inserted immediately after the paragraph within which it is first cited. The picture below is from the Plosone submission guidelines.**

L150 Premature releases are generally detected as the tag staying at a constant depth (+- x meters during y days). This can be due to the factors explained, but also to fish death after tagging and it remaining at the seabed. This does not seem to be the case in the current study, but evaluation of fish mortality based on this (or on the breakage of the pin when depth exceeds a maximum depth to prevent implosion) is frequent in large pelagics satellite tagging studies.

**See lines 147-150: We fully agree with the reviewer. In this sentence we just want to point out what are the most probable causes of premature release. The interpretation of the tags data does not suggest that premature releases were due to fish death, as mentioned by the reviewer. Fish death was added as a possible scenario for early release.**

L160 It is a very remarkable rate, and most times difficult to achieve in the field. How were tags physically recovered, just verifying they had beached and using Argos transmission location or by means of a radiogoniometer?. Consider specifying a bit the procedure.

**See lines 161-163: We added the following sentence “When tags popped-up and began their Argos transmissions, their locations were tracked and a physical retrieval was attempted using a VHF radio, resulting in six successful recoveries. All tags were beached when they were recovered.”**

L163 Consider changing “quality and quantity” by “resolution and coverage”

**See line 166: We followed the reviewer’s suggestion and replaced “quality and quantity” with “resolution and coverage”.**

L173 Transmitted data
**See line 176:We followed the reviewer’s suggestion and replaced “Transmitter” with “Transmitted”**

L184 PDT has not been defined before. I imagine it refers to specific files by WC providing depth-temperature profiles, it should be described.
On the other hand, I am not fully aware on how the depths in the PDT file are generated from the archived records (I guess equally spaced from minimum and maximum depths) but if the hours per histogram is not set at 24 h (i.e., if tags are programmed to summarise data in less than 24 h intervals), depths can be biased low for those days when not all histograms are received. Therefore, some further clarification might be beneficial (e.g., if tags were programmed to summarized TAD, TAT and PDT on a daily basis and the way PDT profiles are generated).

**We thank the reviewer for this comment as the changes made have improved the clarity of the methodology and the ms readability.**

**We made changes to the Materials and Methods (Lines 152-162) to specifically address this comment.**

**“To support the tag’s battery lifespan while transmitting data and to optimise data transmission, data summaries may be collected on a periodical basis via “duty cycling”. This reduces the number of messages the tag stores so that all the messages can be transmitted in packages before the tag battery expires and when satellites are passing over the tag. The tags deployed in 2019 (all 300 days) were scheduled to store time series (depth and water temperature) messages continuously for the first seven days of deployment and then on alternate days (one day on, one day off) whereas summary profile of depth and temperature (PDT) messages were always generated. The tags deployed in 2018 recorded time series summaries continuously, without a schedule but with summary messages of PDT, time at depth (TAD) and time at temperature (TAT) being generated every six hours. Duty cycling did not affect a tag’s full archival dataset, though this is only accessible if the tag (and the archived data) is physically recovered.”**

L221 (table 1) Fish 8 is not noted as having been recaptured.

**Fish 8 was recaptured but well after the study was complete and hence this information was not added to the table as the recapture did not affect the deployment time. the tag was recovered hence bolded and no asterisk.**

L223 The estimated positions refer to the tracks?. If so, some indication on the post-processing (GPE3, WC…) would be desirable. If it refers to tagging and pop-off positions, noting the former is recorded by the taggers and the later is obtained in the argos transmission,with a relatively high accuracy (e.g., less than 350 in class 2 transmissions), I would remove the word “estimated”.

**See lines 227-228: Figure 1 shows the tagging location and the pop-off locations (based on the argos transmissions). We changed the text to reflect this. It now reads “The PSAT tags pop-off locations were restricted to Portugal’s south coast, the Gulf of Cádiz and the Strait of Gibraltar, Spain (Figure 1).“**

L229 Consider changing July and August by early and mid-summer, respectively.

**Changed accordingly. See line 233: “in spring and early summer, and one fish (#8) in summer.”**

L245-246 Construction of this sentence seems a bit strange. Consider beginning with: Fish spent on average 23.2% of their time at temperatures above…

**See lines 249-250: This sentence was replaced with “Fish spent, on average, 23.2% of their time at temperatures above 18ºC and only 1.4% at temperatures below 14º C.”**

L270 The practical totality of what is described in summer months is based on one fish (#20) with just few days from fish #8 in May and July. It is acknowledged in the discussion section, but it should better be also indicated elsewhere, because as it is now it seems to be a conclusion supported by at least several animals.

**This section was rewritten to better accommodate the issue raised by the reviewer. It now reads in lines 275-276: “effects during May and June (based on a single individual - #20) and then again during September and October (data from ten fish).”**

L272 Shallowest water during the middle of the day… it does not seem very clear from figure 4. I can only observe a slight deeper behaviour around dusk, but it seems mostly constant throughout the day… maybe it is due to the colour palette, but might be worth considering it further (again noting they are data from just one fish).

**We believe this might be due to the figure resolution. When reproduced in full resolution the lighter yellow between 10 and 12h is well noticed for the months of May and June, when compared to the darker yellow for night-time hours. We added the information that the May and June data is based on a single individual.**

Line 275-276: “effects during May and June (based on a single individual - #20) and then again during September and October (data from ten fish)”

**Lines 279-280 we added this phase to the end of the sentence: “as this was the only fish where a significant proportion of its tags deployment time was during May and June (Figure S2)”**

Moreover, from figure S2, it seems data were averaged by hour and month, not hour and julian day (every tag data begin or end with the month, not in the middle of it, please double-check). Also, sometimes smoothing masks results, hence, in supplementary information, some raw data can be of help also (ie average depth by hour and julian day).

**Apologise we have just realised that there were two S2 figures, where in fact the reviewer is referring to figure S1 which is a separate PDF to the other supplementary files. This has been corrected but does not change the interpretation of this figure by the reviewer, we unfortunately labled S1 as S2 in the manuscript submission portal.**

**We are however a little confused as the only figure that represents dive depth and julian day is Figure 3.**

**We assume the reviewer is referring to Figure S1 as this is the only figure showing individual contour plots between month and hour for each fish (this was previously listed as Figure S2)**

**Furthermore, the raw data has been plotted in Figure 2**

L291 Note data from most May and June originate from one fish, and in July from two.

**We added the following sentence in lines 297-298: “However, it must be noted that data from May and June is based on a single fish (#20) and from July from two individuals (#8 and #20).”**

L299-300 I would just mention demersal or pelagic behaviour would need to be determined using other methodology (I do see other available methods- e.g acoustic tracking) as more suitable for this objective.

**Following the reviewer’s suggestion this sentence now reads in lines 305-307: “Regrettably, the precise assessment of demersal or pelagic behaviour using the available dataset presents challenges, demanding the exploration of supplementary methodologies, such as employing fish-attached cameras or acoustic tracking.”**

L312 Not sure what standar-tagged refers to. Conventional tags?.

**See line 319: We are referring to acoustic tags. The text was changed accordingly.**

L341-343 Does it mean “In winter, in waters with low stratification, forages to the surface were rare”?
**Rewritten see lines 348-349: “During the winter, when the waters are less stratrified, forages to the surface were rare.”**

L343-344 There are several studies on large pelagic fish (e.g. bluefin tuna) indicating preference to stay over the thermocline particularly at nighttime or when waters are highly stratified.

**We thank the reviewer for pointing this out. Our study species is a demersal coastal species, quite different from an oceanic pelagic species such as the bluefin tuna. We have however added two sentences referring to the similarities between these two species when the thermocline breaks down. See lines 351-355: “Similar findings were found for *Thunnus thynnus* in Mediterranean waters, where fish moved out of shallower waters in the Adriatic Sea when the thermocline broke down at the onset of winter. It must, however, be noted that *A. regius* is a demersal species that is less pelagic in nature when compared to *T. thynnus* and therefore needing to move into shallower coastal waters when the thermocline develops.”**
